# *N*-glycoproteins in Plant Cell Walls: A Survey

**DOI:** 10.3390/plants11233204

**Published:** 2022-11-23

**Authors:** Hélène San Clemente, Elisabeth Jamet

**Affiliations:** Laboratoire de Recherche en Sciences Végétales, Université de Toulouse, CNRS, UPS, Toulouse INP, 31320 Auzeville-Tolosane, France

**Keywords:** cell wall, cell wall protein, Concanavalin A affinity chromatography, glycopeptides, glycoproteomics, hydrophilic interaction liquid chromatography, *N*-glycosylation, plant

## Abstract

Cell walls are an extracellular compartment specific to plant cells, which are not found in animal cells. Their composition varies between cell types, plant species, and physiological states. They are composed of a great diversity of polymers, i.e., polysaccharides, proteins, and lignins. Cell wall proteins (CWPs) are major players involved in the plasticity of cell walls which support cell growth and differentiation, as well as adaptation to environmental changes. In order to reach the extracellular space, CWPs are transported through the secretory pathway where they may undergo post-translational modifications, including *N*-glycosylations on the Asn residues in specific motifs (Asn-X-Ser/Thr-X, with X≠Pro). This review aims at providing a survey of the present knowledge related to cell wall *N*-glycoproteins with (i) an overview of the experimental workflows, (ii) a selection of relevant articles dedicated to *N*-glycoproteomics, (iii) a description of the diversity of *N*-glycans, and (iv) a focus on the importance of *N*-glycans for CWP structure and/or function.

## 1. Introduction

Glycosylations are major post-translational modifications (PTMs) observed in plant cell wall proteins (CWPs), and they occur in the secretory pathway. There are three types: *N*-glycosylation, *O*-glycosylation, and glypiation (for a review, see [1]). *N*-glycosylation consists of the grafting of an oligosaccharide onto a particular amino acid, asparagine (Asn), in the context of a specific motif, Asn-X-Ser/Thr-X (with X≠Pro). This motif is described as PS00001 in the PROSITE database (https://prosite.expasy.org/) (accessed on 22 September 2022) [2]. Since it is short (only four amino acids), this motif is classified among the motifs with a high probability of occurrence. It means that the chance to find it in protein sequences is high even if the protein does not travel through the secretion pathway where *N*-glycans are synthesized and grafted. *O*-glycosylation occurs on the hydroxyl groups of Ser and hydroxyproline (Hyp) residues located in particular amino acid motifs, especially in the structural proteins of the hydroxyproline-rich glycoprotein families (HRGPs) [3,4,5,6,7]. Glypiation consists of the addition of a glycosylphosphatidylinositol (GPI) being anchored at the C-terminus of proteins and allows the anchoring of proteins at the external side of the plasma membrane (for a review, see [8]). All these PTMs are crucial for the structure and the function of CWPs. This review will focus on *N*-glycosylation which affects a great proportion of CWPs.

**Figure 1:**
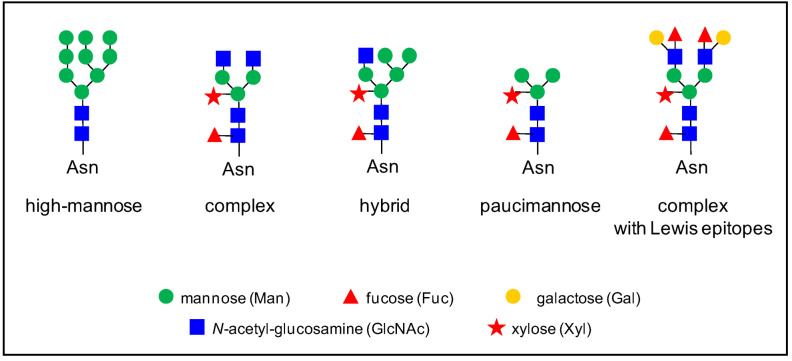
Some examples of *N*-glycan structures grafted onto Asn-X-Ser/Thr motifs (with X≠Pro) of plant *N*-glycoproteins. They all share a core Man_3_GlcNAc_2_ structure. The core structure is substituted by two to six Man residues in high-mannose *N*-glycans. Complex *N*-glycans comprise a β-1,2- linked Xyl, an α-1,3-linked Fuc residue, and/or one or two β-1,2- linked GlcNAc residues bound to the core. Hybrid *N*-glycans share common features with high-mannose and complex *N*-glycans. Paucimannose *N*-glycans do not exhibit terminal GlcNAc residues. Some complex *N*-glycans also comprise one out of the two terminal α-1,4-Fuc and β-1,3-Gal residues which form Lewis a epitopes.

In plants, there are various types of *N*-glycans [9]. The biosynthesis of *N*-glycans has already been extensively reviewed (see for example, [9,10,11,12,13,14]), and it will not be described in detail here. Briefly, *N*-glycosylation process starts in the endoplasmic reticulum (ER) by the co-translational transfer of an oligosaccharide precursor of the high-mannose type, Glc(3)Man(9)GlcNAc(2) (Figure 1), onto the Asn residues of the *N*-glycosylation motifs of proteins. Then, these *N*-glycans undergo several maturation steps consisting of the removal and the addition of sugar residues in the ER and the Golgi apparatus by glycosidases and glycosyl transferases, respectively; this gives rise to complex *N*-glycans with Lewis epitopes. The presence of a core α(1,3)-Fuc and a β(1,2)-Xyl is specific to plant *N*-glycans, compared to mammal *N*-glycans [9]. Overall, different types of *N*-glycans are generated from complex *N*-glycans, such as hybrid and paucimannose *N*-glycans (Figure 1).

The structure of *N*-glycans has evolved in the green lineage [10,11]. In green algae, other types of *N*-glycans have been found. In the microalgae *Chlorella vulgaris*, *N*-glycans are mostly of the high mannose/oligomannose-type, and they are *O*-methylated at their non-reducing termini [15]. In the *Chlamydomonas rheinardtii* green alga, the main *N*-glycans are of three types: oligomannose *N*-glycans represent about 70% of the *N*-glycan population, and complex *N*-glycans with one or two pentose residues account for 14.1 and 16.6%, respectively [16]. In *Penium margaritaceum,* a charophycean green alga assumed to be sister to land plants, the same *N*-glycans as those of flowering plants are found [17]. In the bryophyte *Marchantia polymorpha*, preliminary results indicate the presence of complex *N*-glycans with two pentose residues (H Kolkas et al., unpublished results). In the moss *Physcomitrella patens*, the same types of *N*-glycans as in flowering plants have been characterized [18]. These results suggest that the whole machinery of *N*-glycosylation has been established very early in the green lineage and conserved along the evolution.

The importance of *N*-glycosylation has been stressed by the study of *N*-glycosylation mutants. The severity of their phenotypes depends on which step of *N*-glycan biosynthesis is impaired. The more severe phenotypes are observed when the mutation affects the early steps of *N*-glycan biosynthesis in the ER, especially the biosynthesis of lipid-linked oligosaccharide (LLO) precursors or the multi-subunit oligosaccharyl transferase (OST) complex. Some mutations can be lethal (for reviews, see [13,19]). At later stages of *N*-glycan biosynthesis, mutations can affect post-seedling development, as in the *Oryza sativa N-acetylglucosaminyltransferase 1* (*gnt1*) mutant, but still allow callus development [20]. The *gnt1* mutant is impaired with the addition of the β(1,2)-linked GlcNAc to the core Man(5)GlcNac(2) which is a prerequisite for the synthesis of complex and hybrid *N*-glycans. On the contrary, when the mutation affects a late stage of *N*-glycan biosynthesis, such as the grafting of α(1,3)-Fuc of β(1,2)-Xyl by the core xylosyl transferase (XYLT) or fucosyl transferases (FUT11 and FUT12) in *A. thaliana*, no developmental phenotype has been observed [21]. More precisely, it has been shown that changes in the *N*-glycosylation status of a given protein can affect its biological activity, as exemplified in Section 5 of this review.

We aim at giving an overview of the plant extracellular *N*-glycoproteome by addressing the following points: (i) an overview of the strategies used to isolate *N*-glycoproteins using different experimental workflows (Section 2); (ii) a survey of different *N*-glycoproteomic studies on various plants and organs (Section 3); (iii) a glance at the heterogeneity of *N*-glycans, especially at a given *N*-glycosylation site (Section 4); and (iv) the roles played by *N*-glycans in the structure and function of *N*-glycoproteins (Section 5).

## 2. An Overview of the Strategies

Different strategies have been designed to perform plant glycoproteomic studies. They can be grouped into two sets according to whether the selection of the affinity chromatography of the molecules of interest occurs (i) at the protein level or (ii) at the *N*-glycopeptide level.

**Figure 2:**
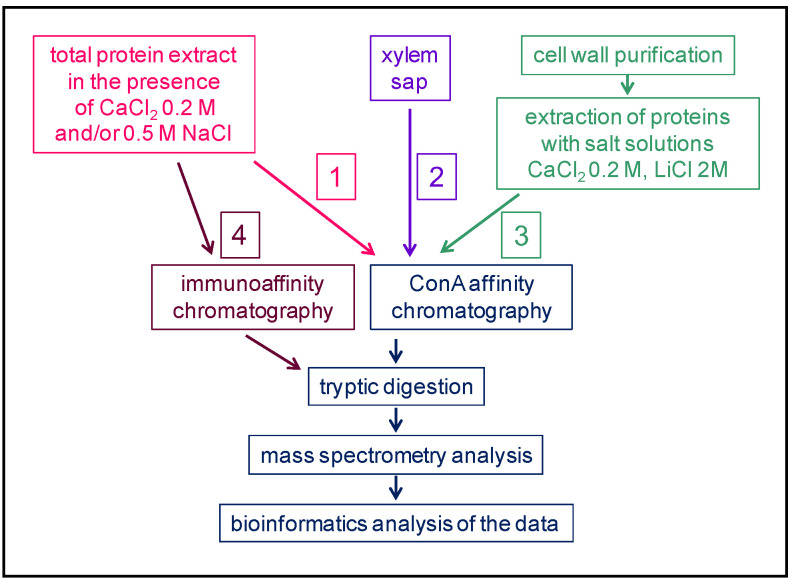
**Schematic representation of different strategies used to study plant *N*-glycoproteomes: the affinity purification step is performed at the protein level on a lectin, Concanavalin A (ConA), in the quoted articles**. Three types of workflow can be distinguished: 1 and 2 start with a total protein extract followed by a ConA affinity chromatography prior to a tryptic digestion, and 3 starts with the purification of a cell wall fraction from which proteins are extracted with salt solutions prior to a tryptic digestion. At the final step, the samples are analyzed using MS and the proteins identified using bioinformatics.

**Figure 3:**
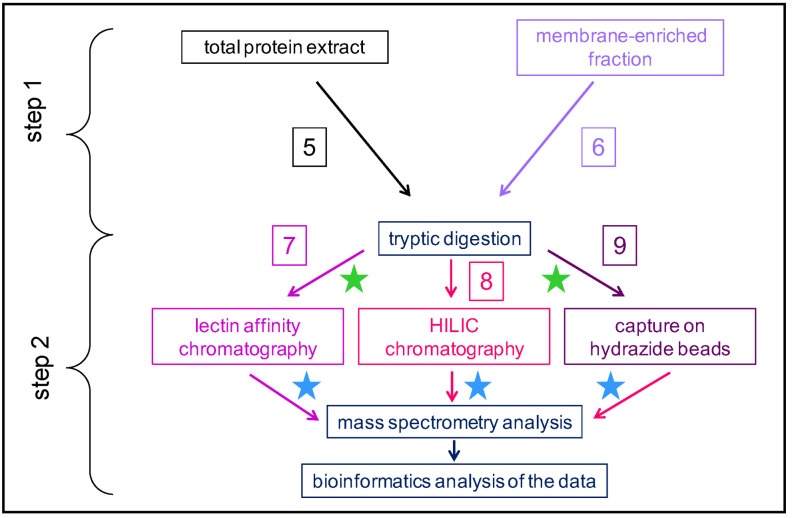
**Schematic representation of different strategies used to study plant *N*-glycoproteomes: the affinity purification step is performed at the peptide level.** Several types of workflow can be distinguished. A total protein extract (workflow **5**) or a membrane-enriched fraction (workflow **6**) can be used as starting points. After tryptic digestion, the enrichment in *N*-glycopeptides can be performed using two kinds of chromatography: a lectin affinity chromatography (workflow **7**) or a HILIC (workflow **8**). *N*-glycopeptides can also be captured on hydrazide beads (workflow **9**). At the final step, the samples are analyzed using MS and bioinformatics. The green stars indicate that a step of peptide labelling can be performed to obtain quantitative data. The blue stars indicate possible treatment with enzymes (e.g., endo H or PNGases) to remove *N*-glycans before the analysis of deglycosylated glycopeptides or *N*-glycans using MS and bioinformatics.

(i) In the first case, there has been three different starting points (Figure 2). Workflow **1** starts with a total protein extract in the presence of salts (e.g., NaCl or CaCl_2_) in order to release the proteins from cell walls [22]. The case of the xylem sap (worflow **2**) is the simplest since the extract is used directly [23]. Workflow **3** involves two steps: The purification of cell walls is followed by the extraction of proteins with salt solutions [24]. Then, a step of enrichment is performed using an affinity chromatography step with Concanavalin A (ConA) lectin of *Canavalia ensiformis* L. (Table 1) [25]. In the case of glycoproteins bearing complex *N*-glycans with Lewis a epitopes, which are rarer and assumed to be specific to growth stages, the Lewis a-specific JIM84 antibodies have been used for immunoaffinity chromatography (workflow **4**) [26]. In all cases, the selected proteins are digested with trypsin and identified using mass spectrometry (MS) and bioinformatics.

(ii) In the second case, the workflows follow two successive steps (Figure 3): step 1 is upstream the tryptic digestion, and step 2 is downstream the tryptic digestion. The starting point can be a total protein extract (workflow **5**) or a membrane-enriched fraction (workflow **6**). The reason for starting with a membrane-enriched fraction is that *N*-glycosylation occurs in the secretion pathway, i.e., in the ER and in the Golgi apparatus. However, the biological relevance of this approach is limited since *N*-glycans may not be fully processed. Then, a tryptic digestion is performed. During the second step, glycopeptides are separated using a lectin affinity chromatography (workflow **7**) or a hydrophilic interaction liquid chromatography (HILIC) (workflow **8**), or they are captured on hydrazide beads (workflow **9**). Different types of lectins have been frequently used: ConA, Wheat germ Agglutinin (WGA), *Galanthus nivalis* Agglutinin (GNA), or *Lens culinaris* agglutinin (LCH). Each of these lectins exhibit different specificities towards the monosaccharides/oligosaccharides forming the *N*-glycans (Table 1). Other types of lectins could be used, as described in detail in [31] In some cases, a multiple affinity chromatography (MLAC) is performed with different lectins to enlarge the coverage of the *N*-glycoproteome [24,32]. As an example, among the 448 proteins identified in a tomato *N*-glycoproteome, 24.5% were specifically captured by ConA, 21.2% by GNA, and 13.2% by LCH [32]. For all these workflows, the last steps are the MS analysis followed by protein identification using dedicated bioinformatics programs.

After tryptic digestion, the labeling of the peptides can be performed by grafting different chemical tags onto their amine groups to obtain quantitative data, allowing the comparison of different experimental conditions, e.g., using the Tandem Mass Tagging (TMT) [33] or the Isobaric Tags for Relative and Absolute Quantitation (iTRAQ) [34] technologies.

**Figure 4:**
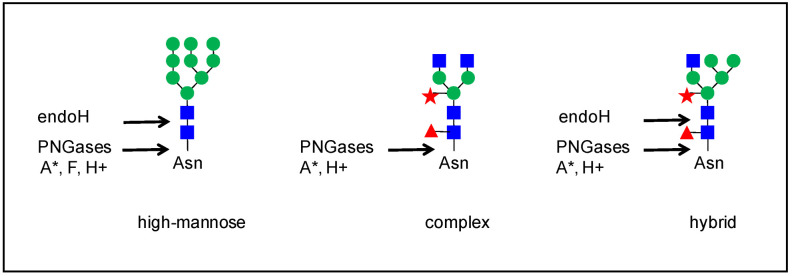
Strategies used to remove *N*-glycans from glycopeptides. The position of the cut is schematized for four enzymes: endoH and PNGAses A, F or H+. Three examples of *N*-glycans are shown (see Figure 1 for a full description). The behavior of paucimannose and complex glycans with Lewis epitopes is similar to that of complex glycans. The star means that PNGase A is more efficient on small peptides.

*N*-glycans can be removed from glycopeptides by an enzymatic digestion with endo-glycosidase H (endo H, from *Streptomyces plicatus*) [26], peptide: *N*-glycosidase (PNGase) A (from almonds) [32,34,35,36], or PNGase F (from *Flavobacterium meningosepticum*) [26,33,34,36,37,38] (Figure 4). Endo-H cleaves the chitobiose core of high mannose and some hybrid *N*-glycans. PNGases have the ability to cleave the bond between the innermost GlcNAc and the Asn residue. However, PNGase F is sensitive to the presence of α-1,3-Fuc on the core GlcNAc, whereas PNGase A is not, but is more efficient on small glycopeptides. PNGase H+ (*from Terriglobus roseus*) combines the properties of PNGases F and A since it is efficient on glycoproteins and on *N*-glycans with core α-1,3-Fuc [39]. However, this enzyme has only been used in a few studies (e.g., [40]). The activity of these enzymes results in the modifications of glycopeptides by converting Asn to Asp in the case of PGNAses and leaving one GlcNAc on the Asn residue in the case of Endo-H [36,41]. This information allows the determination of the occupancy of *N*-glycosylation sites.

It should be noted that some studies have included a fine analysis of *N*-glycan localization and structure using MS. In these cases, *N*-glycans were either studied directly on the glycopeptides [35,38] or they were released by enzymatic digestion with PGNases or Endo-H and subjected to fragmentation in a mass spectrometer [42,43]. The use of Matrix Assisted Laser Desorption Ionization (MALDI-TOF)-MS allows the identification of *N*-glycans on *N*-glycopeptides on the basis of their mass/charge ratio (*m/z*, with z = 1) [35,44], whereas the fragmentation of *N*-glycans using MS-MS allows the reconstitution of their fine structure [32,43]. The fragmentation of a glycopeptide can be performed on the glycan backbone using collision-induced dissociation (CID) or on the peptide backbone using electron transfer dissociation (ETD) [45].

Different types of proteins are identified in these *N*-glycoproteomes with regard to their sub-cellular localization. They can be residents in the ER when they harbor an ER retention signal at their C-terminus, which are described as PS00014 in the PROSITE database [46] and are mainly Lys/Arg/His-Asp-Glu-Leu in plants. However, they can also be located in the Golgi apparatus, in the vacuole, at the plasma membrane, or in the extracellular space, cell wall or apoplasm. In this review, we consider proteins as CWPs when they have a signal peptide predicted by at least two different bioinformatic programs: no ER retention signal and less than two transmembrane domains [47]. This latter criterion was found to be necessary to take into account the presence of a peptide signal at the N-terminus of proteins, which is eventually recognized as a trans-membrane domain since it is hydrophobic, as well as the possible presence of a GPI-anchor signal peptide at the C-terminus. In addition, this criterion takes into account plasma membrane receptors, which are located at the frontier between the extracellular space and the cytosol and are critical for signal transduction. In addition, the predicted functional domains and literature data are considered to assign a protein to a CWP category. To easily obtain these predictions, the *ProtAnnDB* bioinformatics tool can be used (http://www.polebio.lrsv.ups-tlse.fr/ProtAnnDB/) (accessed on 22 September 2022). The main CWP families have been described in previous articles (e.g., [48,49,50,51]). These can be distributed in several classes [48]: proteins acting on cell wall polysaccharides, such as glycoside hydrolases (GHs) or expansins; oxido-reductases, such as class III peroxidases (CIII Prxs); proteases, such as subtilisin Ser proteases; signaling proteins or peptides, such as arabinogalactan proteins (AGPs); proteins related to lipid metabolism, such as non-specific lipid transfer proteins (nsLTPs); proteins with interaction domains, such as protease inhibitors; structural proteins, such as HRGPs; miscellaneous proteins; and proteins of yet unknown function.

## 3. An Overview of the Present *N*-glycoproteomics Studies

A selection of articles devoted to *N*-glycoproteomics is presented in Table 2. Several plants have been studied: *Marchantia polymorpha* [52] as an early divergent plant; *Zea mays* [34], *Triticum aestivum* [33] and *Brachypodium distachyon* [53] as monocot plants; and *Fagopyrum tataricum* [38], *Solanum lycopersicum* [32,37,54], *Gossypium hirsutum* [55], *Camellia sinensis* [56], *Arabidopsis thaliana* [22,24,36,43,57,58] and *Brassica oleracea* [23] as dicot plants. *N*-glycoproteomes have been described in different organs: whole thalli corresponding to the haploid gametophytic stage of *M. polymorpha* [52]; seedlings [59]; aerial organs for flowering plants, such as actively growing hypocotyls of etiolated seedlings [24]; leaves [33,34,43,56]; stems [22,43]; inflorescences [58]; fruit pericarp [32,37,54]; and seeds [38,55]. The *N*-glycoproteome of the xylem sap of *B. oleracea* has also been analyzed, and looks very similar to a cell wall proteome [23]. Additionally, in a few studies, the *N*-glycans released by PNGase A, which cleaves *N*-glycans regardless of the presence of Xyl of Fuc residues, have been analyzed [35,42].

The sizes of these *N*-glycoproteomes are variable, ranging from 62 to 912 identified proteins (Table 2). The final extract which is enriched in *N*-glycoproteins after lectin affinity chromatography or HILIC contains proteins traveling through the whole secretion pathway from the ER to the Golgi apparatus, as well as to membrane proteins and to vacuolar and extracellular proteins. Most of them (from 84% to 100%, Table 2) exhibit the canonical *N*-glycosylation site (Asn-X-Ser/Thr-X, with X≠Pro) (PS00001, https://prosite.expasy.org/) (accessed on 22 September 2022). Some of them contain the canonical C-terminal motif for retention in the ER or are predicted to have trans-membrane domains (see Section 2).

**Figure 5:**
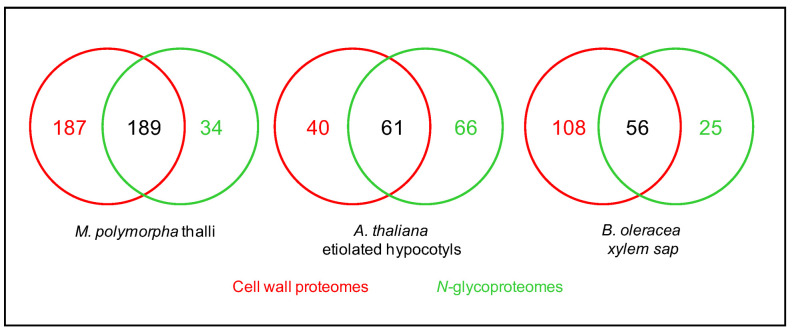
**Comparison between cell wall proteomes** (red) **and *N*-glycoproteomes** (green) **characterized from the same plant material.** In each case, the overall coverage of the cell wall proteome increases by combining the two approaches.

As mentioned above, combining the predictions of sub-cellular localization and functional domains using several bioinformatic programs allows the inference of the cell wall localization of the identified proteins. The percentage of proteins assumed to be CWPs in these *N*-glycoproteomes varies from 55.6 to 98.7% (Table 2). The study of *N*-glycoproteomes, thus, appears as a means to increase the coverage of cell wall proteomes (Figure 5). To illustrate this point, three examples can be mentioned where the same plant material was used to prepare a cell wall proteome from purified cell walls and a *N*-glycoproteome: the thalli of *M. polymorpha* [52], the etiolated hypocotyls of *A. thaliana* [24,60], and the leaves of *C. sinensis* [56]. In the same way, the xylem sap proteome of *B. oleracea* was complemented with a *N*-glycoproteome [23]. These data could be complemented with transcriptomic data for example, but the superimposition of transcriptomic data and cell wall proteomic data has proven to be difficult because of the complexity of the regulation of gene expression at post-transcriptomic levels [61].

## 4. The Heterogeneity of Plant *N*-glycans

All the *N*-glycans studied are grafted on an Asn amino acid residue, but their monosaccharide composition is variable (see Section 1). In Ma et al. [59], 161 possible structures have been collected in a library to allow the identification of *N*-glycopeptides and *N*-glycans in *A. thaliana* seedlings using MS.

**Figure 6:**
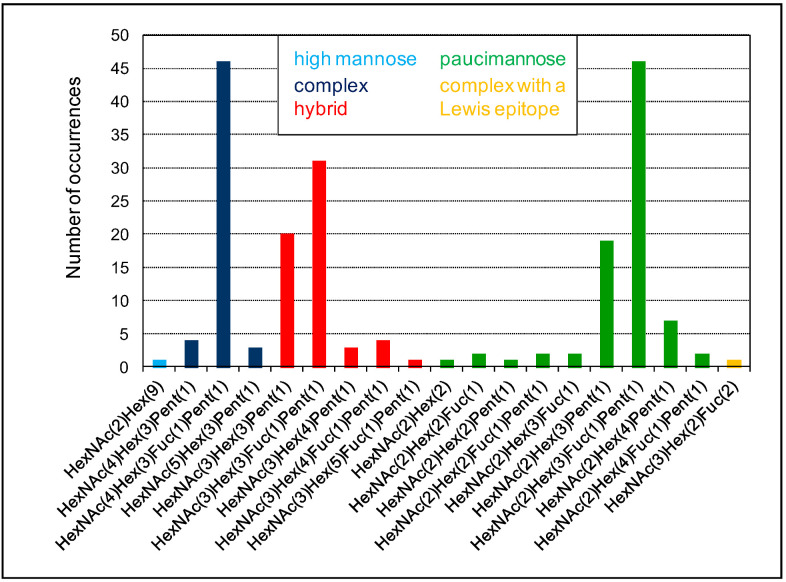
**Diversity of *N*-glycans on a given protein.** This figure uses the MS experimental results related to the At1g68560 protein identified in the *A. thaliana* rosettes *N*-glycoproteome described in [43]. At1g68560 exhibits eight predicted *N*-glycosylation sites, out of which five are shown to be occupied by *N*-glycans. Nineteen different *N*-glycans are found, including a single high mannose-type (light blue), a 53 complex-type (dark blue), a 59 hybrid-type (red), an 82 paucimannose-type (green), and a single complex-type with a Lewis a epitope.

The diversity of *N*-glycans grafted on a given protein is well-illustrated in the case of the *A. thaliana* α-xylosidase (AtXYL1, At1g68560) belonging to the glycoside hydrolase family 31 [62]. Eight *N*-glycosylation sites could be predicted using the Prosite motif (PS00001) on AtXYL1. Among them, five were experimentally found to be occupied [43], and there is no information about the others. Altogether, 19 different *N*-glycans have been identified in AtXYL1 and 196 *N*-glycan occurrences have been observed (Figure 6). These *N*-glycans belong to five classes of *N*-glycans: A single high mannose glycan is found, as well as one complex *N*-glycan with a Lewis a epitope. Otherwise, all the identified *N*-glycans are of the complex-, hybrid-, or paucimannose-type. Major *N*-glycans could be identified in each class: HexNAc(4)Hex(3)Fuc(1)Pent(1) for complex-type (46 occurrences), HexNAc(3)Hex(3)Pent(1) and HexNAc(3)Hex(3)Fuc(1)Pent(1) for hybrid-type (20 and 31 occurrences, respectively), and HexNAc(2)Hex(3)Fuc(1)Pent(1) for paucimannose-type (46 occurrences).

**Figure 7:**
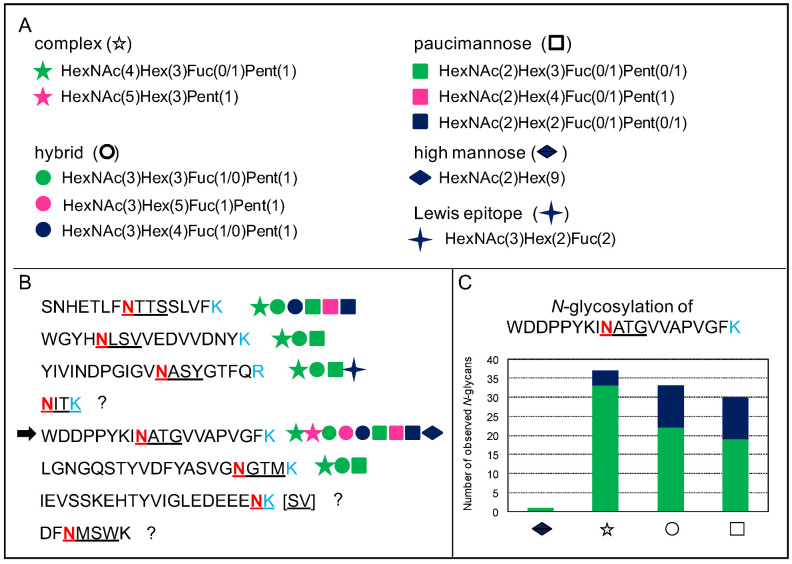
**Microheterogeneity of *N*-glycosylation.** This figure uses the MS experimental results related to the At1g68560 protein identified in the *A. thaliana* rosettes *N*-glycoproteome described in [43]. (**A**). Types of *N*-glycans. Each *N*-glycan type is associated with a symbol. (**B**). Description of the eight predicted *N*-glycopeptides in the amino acid sequence of At1g68560. The Asn (N) residue of the underlined consensus *N*-glycosylation site (PS00001) is in pink, and the tryptic Lys (K) and Arg (R) recognition sites in light blue. In each case, the consensus *N*-glycosylation motif is underlined. *N*-glycans have been found for six out of them. (**C**). Distribution of the different types of *N*-glycans in one of the glycopeptides, as indicated by an arrow in B. The green area corresponds to the major *N*-glycan type in each case, whereas the blue area corresponds to the other types of *N*-glycans: the high mannose-type is represented once by HexNAc(2)Hex(9) (diamond); the major complex-type is HexNAc(4)Hex(3)Fuc(1)Pent(1) (star); the major hybrid-type is HexNAc(3)Hex(3)Fuc(1)Pent(1) (circle); and the major paucimannose-type is HexNAc(2)Hex(3)Fuc(1)Pent(1) (square).

In addition, a given *N*-glycosylation motif can be occupied by different types of *N*-glycans, and this feature has been called microheterogeneity [43]. Figure 7 illustrates this microheterogenity in the case of the At1g68560 protein. Each of the glycosites can be occupied by a great diversity of *N*-glycans as illustrated in Figure 7A,B. In each case, three types of *N*-glycans are always found: hybrid-, complex-, and paucimannose-type. Moreover, for a given glycosite, a major form of each *N*-glycan type is identified. Figure 7C illustrates this point for one of the glycosites: HexNAc(4)Hex(3)Fuc(1)Pent(1) for complex-type; HexNAc(3)Hex(3)Fuc(1)Pent(1) for hybrid-type; and HexNAc(2)Hex(3)Fuc(1)Pent(1) for paucimannose type. In addition to this particular study, this microheterogeneity has been observed for a tomato recombinant protein called XEGIP (xyloglucan-specific endoglucanases inhibitor) produced in *Nicotiana benthamiana* leaves, with a collection of high mannose *N*-glycans (HexNAc(2)(Hex(4-9)) being grafted onto four different *N*-glycosites [32].

Different hypotheses have been proposed to explain this heterogeneity. Since glycosylated proteins travel through the secretion pathway and undergo different steps of *N*-glycan maturation in the ER and the Golgi apparatus [63], they could exhibit different forms of a given *N*-glycan in different cell compartments. In *A. thaliana*, a correlation was found between the predicted sub-cellular localization of the *N*-glycoproteins and their *N*-glycans [43]. For example, high mannose *N*-glycans were found to be predominant in proteins predicted to be localized in the ER, whereas extracellular proteins were found to carry complex, hybrid, and paucimannose *N*-glycan types. Besides, complex *N*-glycans could be degraded by exoglycosidases in the vacuole or in the extracellular compartment [10]. Paucimannose *N*-glycans could be partly degraded by β-*N*-acetylhexosaminidases (HEXO2 and HEXO3) located at the plasma membrane [64] and/or they could be sequentially degraded by bacterial enzymes, such as those of *Xanthomonas campestris* or *Capnocytophaga canimorsus* [65,66]. It was also shown that stress, such as a cold treatment of *A. thaliana* seedlings, could lead to the degradation of high mannose *N*-glycans of proteins located in the ER [59], and that the amount of some glycopeptides was regulated upon de-etiolation of maize seedling leaves [25].

*N*-glycosylation can be regulated at different levels. In *T. aestivum* seedling leaves, the four following motifs were found to be present in plasma membrane-associated proteins and their frequency was found to be modified upon drought stress: Asn-X-Thr (51.2%), Asn-X-Ser (40.0%), Asn-X-Thr-Ala (4.6%), and Ala-Asn-X-Thr (4.2%) with X≠Pro [33]. In *Z. mays* seedling leaves, two motifs were found: Asn-X-Thr (58%) and Asn-X-Ser (42%) [34]. In this study, Ala and Leu were over-represented in the vicinity of both of them. Slightly different proportions of these canonical motifs were found in *B. distachyon* seedling leaves, with 64% of Asn-X-Thr and 36% of Asn-X-Ser [53]. The higher abundance of *N*-glycosylated Asn-X-Thr compared to Asn-X-Ser has been attributed to a higher affinity for the oligosaccharide transferase (OST) [67]. It has also been shown that the amino acid at the X position, as well as the one following the Asn-X-Ser/Thr motif, are important for effective *N*-glycosylation [68,69].

## 5. Roles of *N*-glycans

As mentioned above (see Section 1), the effect of a mutation on a gene encoding a protein involved in the biosynthesis of *N*-glycans can be drastic. However, this is a global effect which does not allow an understanding of the consequences of a modification of *N*-glycosylation status at the level of a given protein. A few examples illustrate how such modifications affect the biological activity of proteins by causing changes in protein solubility or stability, or modifying their sub-cellular localization. This fine-tuning of biological activity is particularly well-demonstrated when a single amino acid mutation destroying a *N*-glycosylation site leads to a reduced level of accumulation of a receptor and the lack of recognition of its ligand [70].

As a first example, it has been shown that the solubility of a CIII Prx of *Coprinus cinereus* can be modulated by increasing its number of *N*-glycans by site-directed mutagenesis, from one *N*-glycosylation and two *O*-glycosylation sites to six *N*-glycosylation sites [71]. The mutated proteins were produced in *Aspergillus oryzae*. They carry up to six high mannose *N*-glycans, as shown using MALDI-TOF MS. It should be mentioned that the enzymatic activity was not modified by the additional *N*-glycosylations. The solubility of the mutant forms of the protein increased in ammonium sulfate solutions with the number of carbohydrate residues in *N*-glycans, and decreased in acetone, showing the importance of *N*-glycans for the solubility of proteins.

The second example is that of a peanut cationic CIII Prx. CIII Prxs are involved in oxido-reduction reactions in plant cell walls. Their activity can result in the non-enzymatic cleavage of polysaccharides by free radicals or the cross-linking of structural proteins or of lignin monomers [72]. The three sites of *N*-glycosylation of this protein, which were experimentally shown to be occupied, have been mutated by changing Asn to Gln in the canonical *N*-glycosylation motifs [73,74]. Three effects were observed: (i) for two mutations (Asn-60-Gln and Asn-144-Gln), the enzymatic activity decreased to 68.6 and 60.0% of that of the wild type enzyme, respectively; (ii) the thermostability of the protein mutated on the Asn-185-Gln residue decreased already at temperatures higher than 40 °C, whereas the two other mutated forms and the wild type were stable up to 55 °C; (iii) the unfolding of the three mutated versions of the protein was quicker in the presence of guanidium chloride, with the protein bearing the Asn-144-Gln mutation being the most affected. Altogether, this study has shown the roles of *N*-glycans in the stability of peroxidase as well as in its catalytic activity.

The third example is that of an *A. thaliana* endo-β1,4-glucanase called KORRIGAN1 (KOR). KOR1 is part of the cellulose synthase complex [75]. It is a plasma membrane protein with eight predicted *N*-glycosylation sites in its extracellular domain. All the *N*-glycosylation sites were shown to be mainly occupied with pauci-mannosidic *N*-glycans when the extracellular domain of KOR was expressed in insect cells [76]. Six *N*-glycosylation sites conserved between different plant species had been mutated (Asn-216/324/345/408/425/567-Gln) [76]. The enzymatic activity was reduced for four of them (Asn-216/324/345/567-Gln). Consistently, the deglycosylation by PNGase F of the wild-type KOR and the mutated versions caused a reduction in the enzymatic activity. When the wild-type KOR1 protein was produced in the presence of kifunensine, an inhibitor of the α-mannosidase involved in the processing of oligomannosidic *N*-glycans in the ER, the enzymatic activity was not affected, thus showing that the processing of *N*-glycans from oligomannosidic to oligonanosidic structures is not required for KOR activity.

Another study on KOR1 has shown the consequences of the modifications of its *N*-glycosylation status on root development [77]. The lack of a single or of several *N*-glycans in the mutated versions of KOR1 has limited effect on root development, whereas the lack of all of them has drastic effects, as shown by the failure to complement the *kor1* mutant (also called *rsw2*). Conversely, the presence of a single *N*-glycan allows at least a partial complementation of the *rsw2* mutant. The best complementation is obtained with *N*-glycans located at the most conserved positions. In addition, the sub-cellular localization of the KOR1 mutant versions had been studied using a confocal microscopy with Green Fluorescent Protein (GFP) fusion proteins [77]. A tonoplast localization could be observed, instead of a plasma membrane localization as observed in the wild-type KOR1. The mutated version devoid of *N*-glycans was mostly trapped into the ER. Altogether these results suggest a role for *N*-glycans in the sub-cellular localization of KOR1.

A fourth example illustrates the role of *N*-glycans in receptor-ligand interactions. The extracellular domain of the *A. thaliana* plasma membrane receptor *EF*-Tu receptor (EFR) exhibits ten *N*-glycosylation sites, among which two that were strictly conserved between related protein sequences were mutagenized to prevent *N*-glycosylation (Asn-143-Gln and Asn-188-Gln) [70]. After transient expression in *N. benthamiana* leaves, the Asn-143-Gln mutation did not allow the interaction with the elf26 ligand, and this elicitation did not lead to the production of reactive oxygen species (ROS) as observed for the wild-type EFR. A decrease in the level of accumulation of the mutated version of the protein was also observed. On the contrary, the protein version carrying the Asn-188-Gln mutation had the same biological activity as the wild-type EFR. These results suggest a role of specific *N*-glycans in protein stability and/or in ligand recognition.

The next example is in regard to the requirement of proper *N*-glycosylation of the Pdi1 disulfide isomerase of *Ustilago maydis* for its full virulence on *Zea mays* [78]. Pdi1 is a protein located in the ER and is involved in the folding of proteins. The *U. maydis pdi1* mutant showed defects in plant infection at the level of fungal expansion. It could be complemented by a wild-type version of Pdi1, but not by a mutated version devoid of *N*-glycans. These experiments, thus, showed that the function of Pdi1 is directly depending on its *N*-glycosylation status.

The last example is taken from the biotechnology field. Indeed, plant cells can be used to produce recombinant proteins for therapeutic uses. The *N*-glycosylation issue is an important one since, although it starts from the same oligosaccharide precursor, the final modifications lead to different types of *N*-glycans (see Section 1). Thus, the plants used for the production of mammal recombinant proteins have to be genetically modified to prevent the grafting of the core α(1,3)-Fuc and β(1,2)-Xyl [79]. In their study, Shin et al. [80] produced the SARS-CoV-2 receptor binding domain in *N. benthamiana* leaves. They prepared three constructs, allowing the production of a wild-type protein carrying three *N*-glycans and two mutants impaired in either one of the two *N*-glycosylation sites (Asn-331-Gln and Asn-343-Gln). The two mutated versions did not accumulate in plant cells unless a human calreticulin lectin chaperone was co-expressed with them. Moreover, the Asn-343-Gln mutant protein was not recognized by a conformation-dependent specific antibody. These experiments show a role of *N*-glycans in protein folding.

## 6. Conclusions

Our knowledge of *N*-glycoproteins has greatly increased over the last years, thanks not only to proteomic and glycomic analyses, but also to studies targeted at specific proteins. The availability of mutants that are impaired in the different steps of *N*-glycan biosynthesis has demonstrated the importance of *N*-glycosylation of proteins for plant development, whereas site-directed mutagenesis of *N*-glycoproteins targeted at their *N*-glycosylation sites has shed light on the precise roles of *N*-glycans. They are indeed critical for protein folding, protein stability, enzymatic activity, sub-cellular localization, or protein–protein interactions, such as recognition by receptors. The reasons for the conservation of *N*-glycosylation sites, the large diversity of *N*-glycan structures, and the variability of *N*-glycosylation site occupancy still have to be fully understood. Another level of heterogeneity has been recently discovered with the in situ localization, using MALDI-TOF MS, of *N*-glycans in the nodules of *Glycine max* formed after infection by *Bradyrhizobium japonicum* [40]. The spatial distribution of *N*-glycans has been found to be uneven, with a more intense *N*-glycosylation in the infected cells and the sclerenchyma layer than in the cortex and the non-infected cells. Tackling the above issues will allow a better understanding of the physiology of plants, and an improvement of the biotechnological processes used for the production of recombinant proteins for therapeutic usages in plant cells.

## Figures and Tables

**Table 1:** Specificities of lectins frequently used for lectin affinity chromatography to capture *N*-glycoproteins or *N*-glycopeptides.

Lectin	Specificity ^a^	Reference
Concanavalin A (ConA)	Man	[25,27]
	branched, terminal α-D-Man	
	terminal β-D-Glc terminal *N*-GlcNAc	
	α-1,2-oligoMan	
Wheat Germ Agglutinin (WGA)	GlcNAc polymer of GlcNAc Sia	[27,28]
	NeuNAc	
*Galanthus nivalis* Agglutinin (GNA)	terminal α-D-Man	[27,29,30]
	α-D-Man-1,3-α-D-Man	
	high Man *N*-glycan	
*Lens culinaris* agglutinin (LCH)	terminal α-D-Man terminal α-D-Glc complex (Man/*N*- GlcNAc core with α-1,6 Fuc)	[27]

^a^ Fuc: fucose; Glc: glucose; Man: mannose; *N*-acetylglucosamine: GlcNAc; NeuNAc: *N*-acetyl neuraminic acid; Sia: sialic acid.

**Table 2:** A selection of *N*-glycoproteomes.

Plant/Green Algae Species	Organ	Strategy ^1^	Size ^2^	Predicted CWPs ^3^	Reference
* Marchantia polymorpha *	thallus ^4^	3	249 (92.0%)	221 (88.8%)	[52]
* Zea mays *	seedling leaf	5/7	476 (100%)	307 (64.5%)	[34]
* Triticum aestivum *	seedling leaf	6/8	312 (100%)	236 (75.6%)	[33]
*Brachypodium distachyon*	seedling leaf	5/7	35 (100%)	28 (80.0%)	[53]
*Fagopyrum tataricum*	seed	5/8	285 (100%)	nd	[38]
*Solanum lycopersicum*	fruit (pericarp) ^4^	5/8	363 (96.7%)	202 (55.6%)	[37]
	fruit (pericarp) ^4^	1	108 (97.2%)	101 (93.5%)	[54]
* Gossypium hirsutum *	seed(fiber cells) ^4^	1	199 (91.5%)	114 (57.3%)	[55]
* Camellia sinensis *	leaf ^4^	5/8	382 (97.9%)	267 (69.9%)	[56]
* Arabidopsis thaliana *	hypocotyl (etiolated) ^4^	3	127 (91.3%)	123 (96.9%)	[24]
	seedling	5/8	912 (nd)	nd	[59]
	seedling/stem/floret	5/8	538 (100%)	343(64%)	[43]
	seedling and leaf^4^	9	173 (84.0%)	135 (78.0%)	[36]
	leaf ^4^	3	62 (98.4%)	58 (93.5%)	[57]
	stem ^4^	1	98 (100%)	88 (89.8%)	[22]
	inflorescence ^4^	5/7	265 (96.6%)	190 (71.7%)	[58]
* Brassica oleracea *	xylem sap ^4^	2	75 (94.7%)	74 (98.7%)	[23]

^1^ See Figure 1 and Figure 2 (Section 2). ^2^ The size corresponds to the total number of identified proteins. The percentages between brackets indicate the proportion of proteins having predicted *N*-glycosylation sites. ^3^ The CWPs are predicted as explained in Section 2. The percentages between brackets indicate the proportion of predicted CWPs in the overall *N*-glycoproteome. ^4^ The data are presented in *WallProtDB-2* (http://www.polebio.lrsv.ups-tlse.fr/WallProtDB/) (accessed on 22 September 2022). Complementary information can be found at SUBA5, but this database is not specifically devoted to CWPs (https://suba.live/) (accessed on 31 October 2022).

## Data Availability

All the data can be found in this article or in *WallProtDB-2* (http://www.polebio.lrsv.ups-tlse.fr/WallProtDB/ (accessed on 31 October 2022)).
